# Characterization of temperature-sensitive leak K^+^ currents and expression of TRAAK, TREK-1, and TREK2 channels in dorsal root ganglion neurons of rats

**DOI:** 10.1186/s13041-018-0384-5

**Published:** 2018-07-06

**Authors:** Viacheslav Viatchenko-Karpinski, Jennifer Ling, Jianguo G. Gu

**Affiliations:** Department of Anesthesiology and Perioperative Medicine, University of Alabama at Birmingham, 901 19TH Street South, BMR II 210, Birmingham, AL 35294 USA

**Keywords:** Dorsal root ganglion neurons, Leak K^+^ currents, Two-pore domain K^+^ channels, Temperature sensitivity, Pain, Cold allodynia

## Abstract

Leak K^+^ currents are mediated by two-pore domain K^+^ (K2P) channels and are involved in controlling neuronal excitability. Of 15 members of K2P channels cloned so far, TRAAK, TREK-1, and TREK-2 are temperature sensitive. In the present study, we show that strong immunoreactivity of TRAAK, TREK-1 and TREK-2 channels was present mainly in small-sized dorsal root ganglion (DRG) neurons of rats. The percentages of neurons with strong immunoreactivity of TRAAK, TREK-1 and TREK-2 channels were 27, 23, and 20%, respectively. Patch-clamp recordings were performed to examine isolated leak K^+^ currents on acutely dissociated small-sized rat DRG neurons at room temperature of 22 °C, cool temperature of 14 °C and warm temperature of 30 °C. In majority of small-sized DRG neurons recorded (76%), large leak K^+^ currents were observed at 22 °C and were inhibited at 14 °C and potentiated at 30 °C, suggesting the presence of temperature-sensitive K2P channels in these neurons. In a small population (18%) of small-sized DRG neurons, cool temperature of 14 °C evoked a conductance which was consistent with TRPM8 channel activation in cold-sensing DRG neurons. In these DRG neurons, leak K^+^ currents were very small at 22 °C and were not potentiated at 30 °C, suggesting that few temperature-sensitive K2P channels was present in cold-sensing DRG neurons. For DRG neurons with temperature-sensitive leak K^+^ currents, riluzole, norfluoxetine and prostaglandin F2α (PGE2α) inhibited the leak K^+^ currents at both 30 °C and 22 °C degree, and did not have inhibitory effects at 14 °C. Collectively, the observed temperature-sensitive leak K^+^ currents are consistent with the expression of temperature-sensitive K2P channels in small-sized DRG neurons.

## Introduction

K2P channel is a family of 15 members that form what is known as “leak K^+^ channels” [8, 10, 18]. Leak K^+^ channels are so called because they constitutively open to yield “leak K^+^ currents” without noticeable voltage-gating properties in conventional electrophysiological recording experiments. K2P channels are present in different tissues including neurons, and in neurons they are involved in setting resting membrane potentials [8]. Of the 15 K2P subtypes, TRAAK, TREK-1, and TREK-2 are 3 subtypes highly relevant to sensory functions due to their high mechanical and thermal sensitivity. These three channels are thus termed mechanothermal K2P channels or thermal K2P channels [8, 13, 20]. Thermal K2P channels are also highly sensitivity to lipids including arachidonic acid [9, 16], polyunsaturated fatty acids, and lysophospholipids [8], and to intracellular and extracellular pH [4, 16, 21].

The expression of thermal K2P channels in somatosensory neurons has been studied by using RT-PCR technique. It has been shown that mRNAs of TREK-1 channels are present in rat dorsal root ganglia (DRG) and their levels are increased after partial bladder outlet obstruction [27]. TREK-1 expression is also increased in mouse DRGs following chronic constriction nerve injury [11]. Thermal K2P channels are found to be upregulated in fast blue-labeled thoracolumbar and lumbosacral somata after inflaming the prostate with intraprostate injection of zymosan [26]. Thermal K2P channels are shown to be expressed in the majority of DRG neurons innervating the mouse colon. The expression of these channels becomes decreased in mouse colon sensory neurons in colitis [17]. Functionally, thermal K2P channels have been suggested to control both warm and cold perceptions [24] and polymodal pain perceptions [2]. By examining leak K^+^ channel mRNAs in DRGs, a previous study also provides evidence suggesting that changes of these channel expression may be involved in spontaneous pain behavior in tissue inflammation [22]. Immunoreactivity of TREK-2 is found to be selectively present in IB4-binding C-fiber nociceptors of rats and these channels are shown to limit spontaneous pain [1]. However, expression of TRAAK, TREK-1 and TREK-2 proteins has not been characterized and compared together in rats L5 DRGs. Furthermore, previous studies on temperature sensitivity of these K2P channels are mainly performed on cloned channels expressed in heterologous expression system. Temperature sensitivity of isolated leak K^+^ currents that are mediated by thermal K2P channels has not been characterized in rat DRG neurons. Pharmacologically, thermal K2P channels expressed on heterologous expression system could be affected by compounds including riluzole, norfluoxetine [6, 9, 15, 18]. However, effects of these compounds on temperature-sensitive leak K^+^ currents have not been characterized in rat DRG neurons. Thermal K2P channels may be also regulated by ligands of G-protein coupled receptors [8]. In the present study, we used immunochemistry method to examine expression of TRAAK, TREK-1 and TREK-2 in neurons of L5 dorsal root ganglions of rats. We further studied, using acutely dissociated lumbar DRG neurons and the patch-clamp recording technique, temperature sensitivity of leak K^+^ currents and effects of riluzole, norfluoxetine and prostaglandin F2α (PGF2α) on temperature-sensitive leak K^+^ currents.

## Methods

Animal care and use conformed to NIH guidelines for care and use of experimental animals. Experimental protocols were approved by the Institutional Animal Care and Use Committee of the University of Alabama at Birmingham.

### Immunohistochemistry

Male rats at ages of 5 to 8 weeks were anesthetized with ketamine/xylazine (100 mg/kg:10 mg/kg, *i.p.*), transcardially exsanguinated with 0.1 M phosphate buffered solution (PB), and perfused with 4% paraformaldehyde (PFA) in 0.1 M PB solution. DRGs at lumbar 5 (L5) levels were removed and placed in a 30% sucrose solution for cryoprotection for two nights. The DRGs were then mounted with a tissue freezing medium (TFM-5, ThermoFisher Scientific, Waltham, MA USA) on a metal tissue holder and immediately snap-frozen by covering the DRGs with dry ice powder. The DRGs were cut on a cryostat (Leica Biosystems, Buffalo Grove, IL, USA) into 10-μm sections. The sections of DRGs were thaw-mounted onto slides and allowed to air-dry for 25 min. DRG sections were then encircled with hydrophobic resin (PAP Pen, The Binding Site). The slide-mounted sections were rinsed with the BupHTM Modified Dulbecco’s Phosphate Buffered Saline (PBS, ThermoFisher Scientific, Waltham, MA USA) 3 times, and then sequentially incubated at room temperature with ethanol solutions at the concentrations of 50% for 10 min, 70% for 10 min and 50% for 10 min. The slides were rinsed 3 times with PBS and sections incubated with a blocking solution containing 10% normal goat serum in PBS for 30 min at room temperature. The sections were incubated with a primary antibody (in 5% normal goat serum in PBS) at 4 °C for 1 night. Primary antibodies include polyclonal rabbit anti-TRAAK antibody (1:2000, APC-108, Alomone labs), polyclonal rabbit anti-TREK-1 antibody (1:2000, APC-108, Alomone labs), and polyclonal rabbit anti-TREK2 antibody (1:2000, APC-055, Alomone labs). Following 3 rinses with PBS solution, the sections were incubated with a secondary antibody for 1 h at room temperature. The secondary antibody (1:1000 in 5% normal goat serum in PBS) was a goat anti-rabbit IgG conjugated with Alexa-594. The sections were rinsed 3 times with PBS solution, cover-slipped with the Prolong Diamond Antifade Mountant medium (ThermoFisher Scientific). Slices were viewed under an upright fluorescent microscope (BX-43, Olympus, Tokyo, Japan) and images were captured using a CCD camera (DP80, Olympus, Tokyo, Japan).

### Patch-clamp recordings

Male rats at ages of 5–8 weeks old were euthanized by overdose of isoflurane. DRGs (L4-L6) were dissected out and harvested from these rats. DRGs were then incubated with dispase II (5 mg/ml) plus type I collagenase (2 mg/ml) in 2 ml Krebs bath solution at 34 °C for 45 min. The Krebs solution contained (in mM) 145 NaCl, 5 KCl, 2 MgCl_2_, 2 CaCl_2_, 5.5 glucose, and 10 HEPES, pH of 7.35, osmolarity of 330 mOsM. After a rinse, DRGs were triturated to dissociate the neurons in the Krebs bath solution, and the dissociated DRG neurons were plated on glass coverslips coated with poly-D-lysine (Sigma) and maintained at room temperature in Krebs solution. Whole-cell patch-clamp recordings were performed within 1 to 4 h after cell plating. Patch-clamp recordings for isolated leak K^+^ currents were performed under the whole-cell mode with recording electrode internal solution contained (in mM): 70 Cs_2_SO4, 5 KCl, 2.4 MgCl_2_, 0.5 CaCl_2_, 5 EGTA, 10 HEPES, 5 Na_2_ATP, 0.33 NaGTP, pH 7.35 and osmolarity of 330 mOsm. The bath solution for isolating leak K^+^ currents contained (in mM): 145 Choline-Cl, 5 KCl, 2 MgCl_2_, 20 TEA-Cl, 10 HEPES, 10 Glucose, pH 7.35 and osmolarity 330 mOsm. Cells were perfused with the bath solution flowing at 1 ml/min in a 0.5 ml recording chamber placed on the stage of an Olympus IX70 microscope. Recording electrode resistance was ~ 5 MΩ. The junction potential was 14 mV and was adjusted during data analysis. The series resistance of each recording was below 30 MΩ and was not compensated. Recording signals were amplified with Axopatch 200B (Axon Instruments), filtered at 2 kHz, and sampled at 5 kHz using pClamp 10 software (Axon Instruments). Unless otherwise indicated, all reagents were purchased from Sigma. To determine leak K^+^ currents following voltage steps, cells were held at − 74 mV under voltage-clamp configuration, and voltage steps were applied from − 104 mV to 34 mV with increments of 10 mV each step and step duration of 500 ms. To determine temperature sensitivity of leak K^+^ currents, recordings were performed at the room temperature of 22 °C, cool temperature of 14 °C and warm temperature of 30 °C. Pharmacology of leak K^+^ currents in acutely dissociated DRG neurons were tested with compounds including norfluoxetine (5 μM), riluzole (100 μM) and PGE2α (1 μM). After recordings of leak K^+^ currents in the absence of testing compounds, the testing compounds were bath applied separately to the recorded cells for 10 min and recordings were then performed in the presence of the testing compounds.

### Data analysis

For the data of immunostaining on L5 DRG sections, immunoreactivity-positive and immunoreactivity-negative neurons in L5 DRG sections were counted and analysed offline from the acquired fluorescent images. For electrophysiological data, leak K^+^ currents were analyzed using Clampfit 10 software. Total leak currents included both leak K^+^ currents and leak currents of electrode-membrane seals. In calculating leak K^+^ currents, the leak currents of electrode-membrane seals were subtracted from total leak currents. Input resistance was calculated based on the steady state currents following a 10-mV voltage step from − 74 mV to − 64 mV. Unless otherwise indicated, data are reported as mean ± SEM, *p < 0.05, **p < 0.01, and ***p < 0.001, paired Student’s t test or one way ANOVA with Dunnett post hoc test.

## Results

**Fig. 1:**
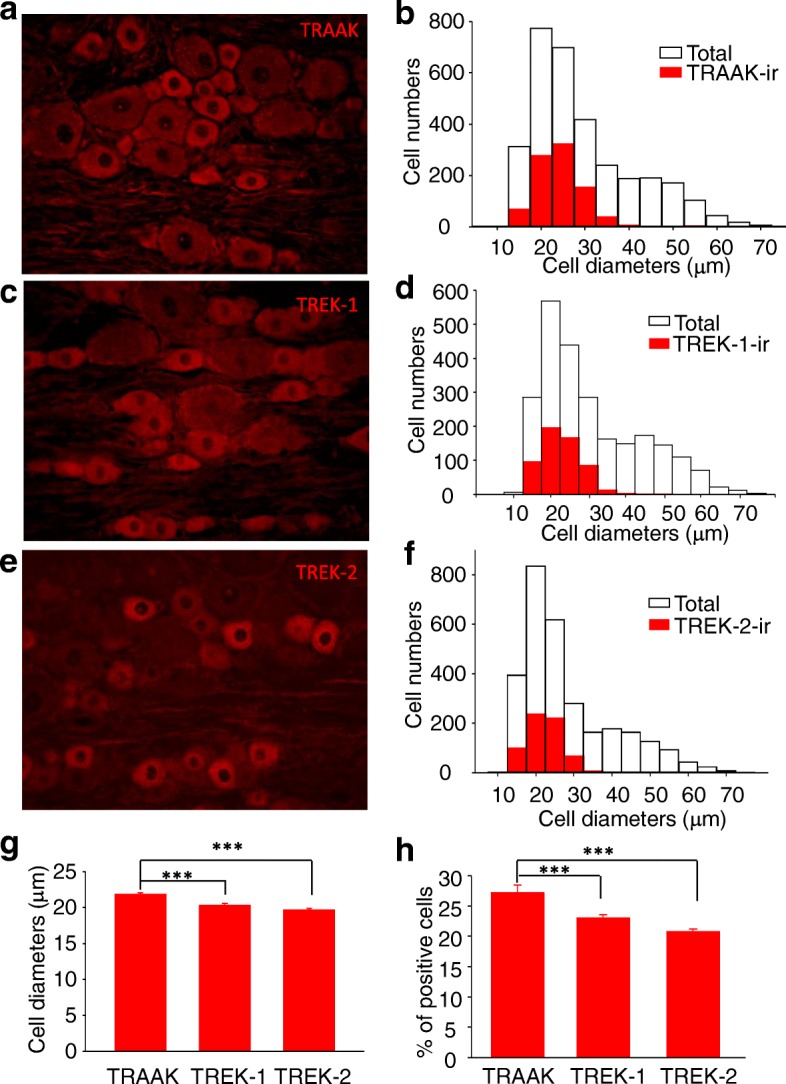
Immunoreactivity of thermal K2P channels in lumbar dorsal root ganglia of rats. **a** Image shows TRAAK immunoreactivity (TRAAK-ir) in a L5 DRG section from an adult rat. **b** Overlay histograms show size distribution of TRAAK-ir positive (red bars, *n* = 890 cells) and total cells (open bars, *n* = 3178 cells). **c** Image shows TREK-1 immunoreactivity (TREK-1-ir) in a L5 DRG section from an adult rat. **d** Overlay histograms show size distribution of TREK-1-ir positive (red bars, *n* = 574 cells) and total cells (open bars, *n* = 2430 cells). **e** Image shows TREK-2 immunoreactivity (TREK-2-ir) in a L5 DRG section from an adult rat. **f** Overlay histograms show size distribution of TREK-2-ir positive (red bars, *n* = 630 cells) and total cells (open bars, *n* = 2928 cells). **g** Mean diameters of cells being positive to TRAAK-ir (1st bar, *n* = 890 positive cells), TREK-1-ir (2nd bar, *n* = 574 positive cells) and TREK-2-ir (3rd bar, *n* = 630 positive cells). **h** Percent of cells being positive of TRAAK-ir (1st bar, *n* = 8 sections), TREK-1-ir (2nd bar, *n* = 7 sections) and TREK-2-ir (3rd bar, *n* = 8 sections). Data in G and H represent Mean ± SEM, ***p < 0.001

We examined expression of TRAAK, TREK-1 and TREK-2 on L5 DRG neurons of rats using immunohistochemistry method (Fig. 1). Strong TRAAK immunoreactivity (TRAAK-ir) was observed primarily in small-sized (< 35 μm) L5 DRG neurons (Fig. 1a and b). Although a small number of medium-sized L5 DRG neurons (35–45 μm) also showed strong TRAAK-ir, most of medium- and large-sized (> 45 μm) L5 DRG neurons showed very weak immunoreactivity. Nevertheless, the weak TRAAK-ir in these medium to large-sized DRG neurons appeared to be higher than the background level (Fig. 1a). Similar to TRAAK-ir, strong TREK-1 immunoreactivity (TREK-1-ir) was mainly observed in small-sized L5 DRG neurons (Fig. 1c and d). In contrast, medium- and large-sized L5 DRG neurons showed very weak immunoreactivity which appeared to be slightly higher than the background level (Fig. 1c). Strong TREK-2 immunoreactivity (TREK-2-ir) was observed exclusively in small-sized L5 DRG neurons (Fig. 1e and f). Medium- to large-sized L5 DRG neurons were shown to be negative of TREK-2-ir. Overall, the sizes of TRAAK-ir, TREK-1-ir and TREK-2-ir L5 DRG neurons were 21.8 ± 0.19 (*n* = 890 cells), 20.3 ± 0.23 (*n* = 574), and 19.7 ± 0.18 (*n* = 630) μm (Fig. 1g). Percentages of TRAAK-ir, TREK-1-ir and TREK-2-ir L5 DRG were 27.2 ± 1.24% (*n* = 8 sections), 23.1 ± 0.52% (*n* = 7 sections), and 20.1 ± 0.38% (*n* = 8 sections) in L5 DRG neurons, respectively (Fig. 1h).

**Fig. 2:**
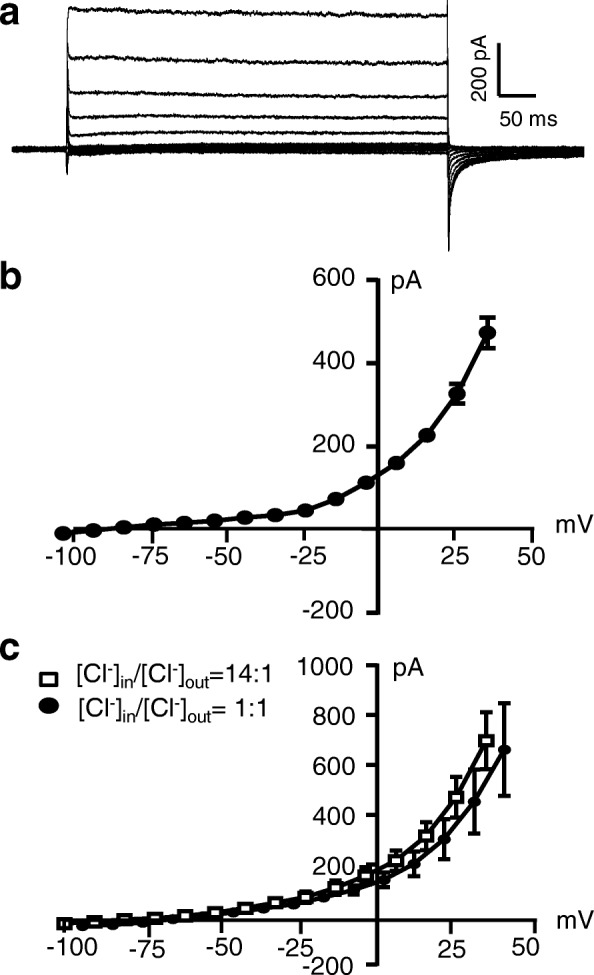
Determination of isolated leak K^+^ currents in acutely dissociated small-sized DRG neurons of rats. **a** Sample traces show isolated leak currents recorded from an acutely dissociated small-sized DRG neuron following voltage-steps from − 104 mV to 34 mV with increments of 10 mV each step. Recordings were performed at the room temperature of 22 °C. **b** I-V relationship of the leak currents recorded at 22 °C from acutely dissociated small-sized DRG neurons (*n* = 42). **c** I-V curve of leak currents recorded with ratio of intracellular and extracellular Cl^−^ concentrations being 14:1 (*n* = 9) and 1:1 (*n* = 8). Data represent Mean ± SEM in (**b**) and (**c**)

We performed patch-clamp recordings from small-sized neurons acutely dissociated from L4–6 DRGs. To isolate leak K^+^ currents, Cs^+^-based recording electrode internal solution was used with Cs^+^ ion being a blocker for voltage-gated K^+^ channels and also being a cation permeable to K2P channels. Furthermore, our bath solution (extracellular side) contained TEA to block voltage-gated K^+^ channels, and the bath solution contained no Na^+^ and Ca^2+^ to null the currents flowing through voltage-gated Na^+^ and Ca^2+^ channels. The ionic condition would yield a reversal potential near − 80 mV for cations flowing through K2P channels. With these ionic conditions and under the voltage-clamp conditions, we observed non-inactivating currents following the application of voltage-steps to the recorded DRG neurons (Fig. 2a). The non-inactivating currents showed outward rectification and had reversal potentials around − 75 mV (Fig. 2a and b), near the reversal potentials of thermal K2P channels. Cl^−^ conductance did not appear to significantly contribute to the non-inactivating currents under our recording conditions since changing ratio of extracellular and intracellular Cl^−^ concentrations did not have significant effects on the reversal potentials of the non-inactivating currents (Fig. 2c). These results indicating that the non-inactivating currents recorded under our experimental conditions were isolated leak K^+^ currents in DRG neurons.

**Fig. 3:**
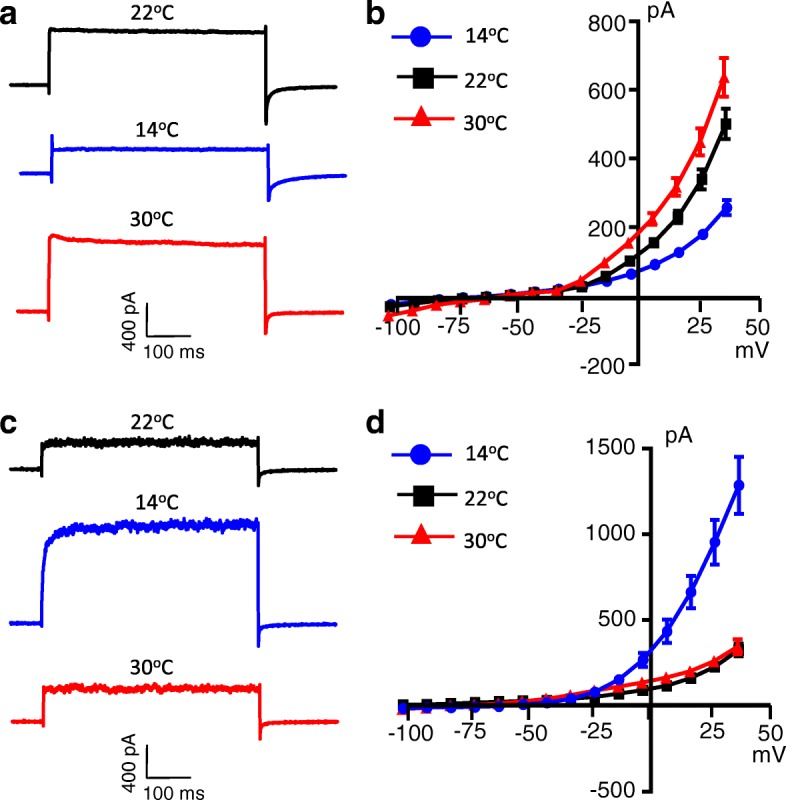
Temperature sensitivity of isolated leak K^+^ currents in acutely dissociated small-sized DRG neurons of rats. **a** Sample traces of isolated leak K^+^ currents recorded from an acutely dissociated small-sized DRG neuron at room temperature of 22 °C (top), cool temperature of 14 °C (middle), and warm temperature of 30 °C (bottom). The leak currents were recorded at the voltage step from − 74 mV to 34 mV. **b** I-V curves of the temperature sensitive leak K^+^ currents illustrated in (**a**) (*n* = 32). **c** Sample traces of leak K^+^ currents recorded from an acutely dissociated small-sized DRG neurons at room temperature of 22 °C (top) and warm temperature of 30 °C (Bottom). Cool temperature of 14 °C evoked a conductance (Middle) which was consistent with TRPM8 activation. Currents were recorded at the voltage step from − 74 mV to 34 mV. **d** I-V curves of leak K^+^ currents at 22 °C and 30 °C as well as conductance at 14 °C illustrated in (**c**) (*n* = 10). Data represent Mean ± SEM in (**b**) and (**d**)

We examined effects of temperatures on isolated leak K^+^ currents in small-sized L5 DRG neurons. Two general types of cells were encountered based on their temperature sensitivity in these small-sized L5 DRG neurons. In the first type of cells, large leak K^+^ currents were recorded at room temperature of 22 °C, and the leak K^+^ currents were strongly inhibited at cool temperature of 14 °C and potentiated at warm temperature of 30 °C (Fig. 3a and b). Of 42 cells tested, ~ 76% of them (32 out of 42 cells) displayed the temperature-sensitive leak K^+^ currents (Fig. 3b, *n* = 32). Consistent with temperature sensitivity of the leak K^+^ currents, input resistance of these cells showed significant increases at cool temperature of 14 °C (*n* = 32, *p <* 0.001) and a strong tendency of decreases at warm temperature of 30 °C (*n* = 32, *p* = 0.08) when comparisons were made with those at 22 °C (Table 1). In the second types of cells, leak K^+^ currents were very small at room temperature of 22 °C and were not increased by warm temperature of 30 °C (Fig. 3c and d, *n* = 10). However, cool temperature of 14 °C evoked a conductance that could be only explained by the activation of the cold-sensing TRPM8 channels (Fig. 3c and d). These cells were also found to have significantly smaller membrane capacitance in comparison with the first type of cells (Table 1). Cool temperature of 14 °C and warm temperature of 30 °C had no significant effects on input resistance in these cells (Table 1). Therefore, few temperature-sensitive leak K^+^ current was present in these cold-sensing DRG neurons.

**Table 1:** Temperature sensitivity of input resistances in acutely dissociated small-sized DRG neurons that display temperature-sensitive leak K^+^ currents

	Membrane capacitance (pF) at 22 °C	Input resistance (GΩ)		
		*T* = 14 °C	*T* = 22 °C (control)	*T* = 30 °C
Type 1 (*n* = 32)	22.93 ± 1.47	2.85 ± 0.41*	1.99 ± 0.34	1.42 ± 0.3^p = 0.08^
Type 2 (*n* = 10)	10.88 ± 1.67^#^	3.24 ± 0.7^ns^	2.93 ± 0.74	2.48 ± 0.72^ns^

Data represent Mean ± SEM, *p < 0.05, *ns* not significantly different, comparing with the values at 22 °C; ^#^*p* < 0.05, comparing between type 1 and type 2 cells

**Fig. 4:**
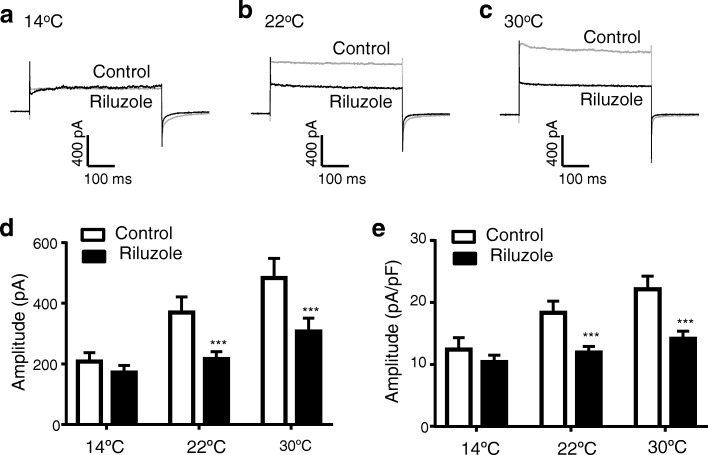
Effects of riluzole on isolated leak K^+^ currents at different temperatures in acutely dissociated small-sized DRG neurons of rats. **a**–**c** Three sets of sample traces show isolated leak K^+^ currents at 14 °C (**a**), 22 °C (**b**) and 30 °C (**c**). In each set of overlaying traces, gray line is isolated leak K^+^ current in the absence and black line is isolated leak K^+^ current in the presence of 100 μM riluzole. **d** Summary data of the amplitudes of isolated leak K^+^ currents at different temperatures in the absence (control, open bars) and present of 100 μM riluzole (closed bars, *n* = 14). **e** Summary data of the normalized amplitudes of isolated leak K^+^ currents at different temperatures in the absence (control, open bars) and present of 100 μM riluzole (closed bars, *n* = 14). In all recordings, leak K^+^ currents were induced by voltage steps from − 74 mV to 34 mV. Data represent Mean ± SEM, *p < 0.05, **p < 0.01, comparing between controls and in the presence of riluzole, two-way ANOVA

**Fig. 5:**
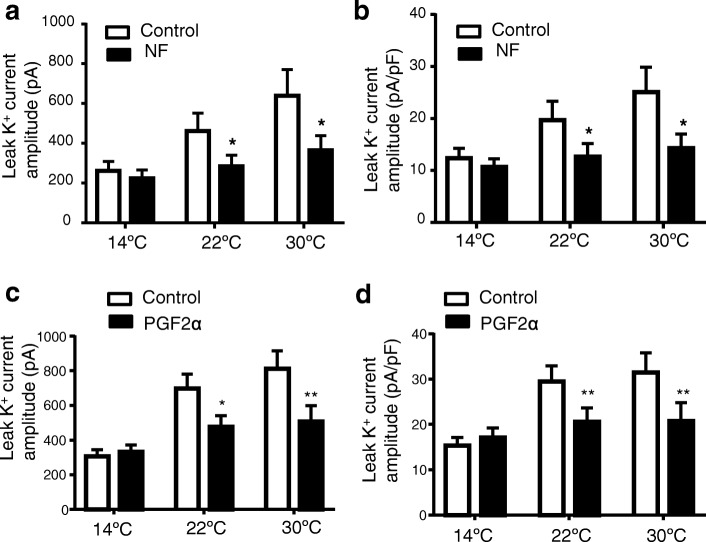
Effects of norfluoxetine and PGF2α on isolated leak K^+^ currents at different temperatures in acutely dissociated small-sized DRG neurons of rats. **a** Summary data of the amplitudes of isolated leak K^+^ currents at 14 °C, 22 °C and 30 °C in the absence (control, open bars) and present of 5 μM norfluoxetine (NF, closed bars, *n* = 7). **b** Summary data of the normalized amplitudes of isolated leak K^+^ currents at 14 °C, 22 °C and 30 °C in the absence (control, open bars) and present of 5 μM NF (closed bars, *n* = 7). **c** Summary data of amplitudes of isolated leak K^+^ currents at 14 °C, 22 °C and 30 °C in the absence (control, open bars) and present of 1 μM PGE2α (closed bars, *n* = 8) **d** Summary data of normalized amplitudes of isolated leak K^+^ currents at 14 °C, 22 °C and 30 °C in the absence (control, open bars) and present of 1 μM PGE2α (closed bars, *n* = 8). In all recordings, leak K^+^ currents were induced by voltage steps from − 74 mV to 34 mV. Data represent Mean ± SEM, *p < 0.05, **p < 0.01, comparing between control and in the presence of the testing compounds, two-way ANOVA

For the first type of cells with temperature-sensitive leak K^+^ currents, we determined effects of riluzole, norfluoxetine and PGE2α on leak K^+^ currents at cool temperature of 14 °C, room temperature of 22 °C, and warm temperature of 30 °C. At 14 °C, leak K^+^ currents were already suppressed by the cool temperature and there was no further significant effect by riluzole (100 μM) on leak K^+^ currents (Fig. 4a, d, e). However, at 22 °C and 30 °C, leak K^+^ currents were larger and riluzole significantly inhibited the leak K^+^ currents at both temperatures (Fig. 4b-e). Similar to riluzole, norfluoxetine (5 μM) also significantly inhibited leak K^+^ currents at the temperatures of 22 °C and 30 °C but had no significant effects at 14 °C (Fig. 5a and b). Of 11 cells tested with PGF2α (1 μM), leak K^+^ currents in 8 cells were significantly inhibited by PGF2α at 22 °C and 30 °C but not affected by PGF2α at 14 °C (Fig. 5c and d), and the leak K^+^ currents in remaining 3 cells were not affected by PGF2α at any temperatures.

## Discussion

A previous study suggested that TREK-2 channel is a major leak K^+^ channels in DRG neurons [14]. A more recent study shows that TREK-2 channels are expressed selectively in IB4-binding C-fiber nociceptors [1]. In the present study we show that strong immunoreactivity to TRAAK and TREK-1 is present in many small-sized DRG neurons and in a small number of medium-sized DRG neurons. Most medium- to large-sized DRG neurons appeared to have weak immunoreactivity to TRAAK and TREK-1. In contrast, immunoreactivity to TREK-2 is exclusively present in small-sized DRG neurons, and medium- to large-sized DRG neurons are lack of immunoreactivity to TREK-2. When cells with strong immunoreactivity are counted, it appears that TRAAK, TREK-1 and TREK-2 channels are expressed primarily in small-sized L5 DRG neurons of rats. However, the possible expression of TRAAK and TREK-1 in many medium- to large-sized DRG neurons cannot be excluded because of their weak immunoreactivity on these cells. Our result of TREK-2 expression in small-sized L5 DRG is consistent with TREK-2 expression in C-fiber nociceptors shown in the previous study [1]. We further show that the order of expression abundance is TRAAK>TREK-1 > TREK-2 in L5 DRG neurons. The expression of TRAAK, TREK1 and TREK-2 in small-sized DRG neurons raises a high possibility that they may be co-expressed in the same neurons to mediate leak K^+^ currents.

We performed patch-clamp recordings of isolated leak K^+^ currents on small-sized DRG neurons acutely dissociated from adult rats. To our knowledge this is the first study of isolated temperature-sensitive leak K^+^ currents on somatosensory neurons. Our results of temperature-sensitive leak K^+^ currents are consistent with the expression of TRAAK, TREK-1 and TREK-2 channels in many small-sized L5 DRG neurons shown by our immunohistochemistry method. We show that leak K^+^ currents in majority of small-sized DRG neurons were decreased by cool temperature of 14 °C and increased by warm temperature of 30 °C. This suggests a significant contribution of temperature-sensitive K2P channels in membrane leak conductance in these sensory neurons. We have previously shown that cool temperatures significantly decrease action potential rheobase and increase the excitability of nociceptive-like somatosensory neurons, and the excitatory effects were accompanied by a significant increase of membrane input resistance [12]. The excitability changes at cool temperatures shown in our previous study may be explained by the effects of cool temperatures on temperature-sensitive K2P channels demonstrated in the present study.

In a small population of small-sized DRG neurons cool temperature of 14 °C evoked a conductance that was consistent with TRPM8 channel activation [23, 25]. Since TRPM8 is a non-selective cation channel, its activation should mainly produce outward currents under the ionic conditions of our recordings. Interestingly, we found that leak K^+^ currents were very small and not temperature sensitive in these cold-sensing neurons. Our results suggest that few thermal sensitive K2P channel is expressed in cold-sensing TRPM8-expressing DRG neurons. This would further suggest that temperature-sensitive K2P channels have minimal role in controlling the excitability of cold-sensing TRPM8-expressing DRG neurons.

For temperature-sensitive leak K^+^ currents in small-sized DRG neurons, we show that riluzole, norfluoxetine and PGF2α significantly inhibited leak K^+^ currents at 22 °C and 30 °C. However, the leak K^+^ currents recorded at 14 °C were not further inhibited in these cells by the three compounds. These results may suggest that cold temperatures of 14 °C maximally inhibited the thermal K2P channels. Therefore, the inhibitory effects of the cool temperature occluded the inhibitory effects of these compounds. In heterologous expression system, riluzole produces phasic potentiation followed by inhibition of TREK-1 and TREK-2 channels, and it also produces tonic potentiation of TRAAK channels; norfluoxetine inhibits both TREK-1 and TREK2 channels [7, 18]. Effects of PGF2α on leak K^+^ channels have not been reported previously but its derivative 11-deoxy-PGF2α has been shown to inhibit TREK-1 and activate TREK-2 channel [5]. We show that all three compounds produced inhibitory effects on temperature-sensitive leak K^+^ currents. This may suggest that temperature-sensitive leak K^+^ currents in these cells are mediated by two or more temperature-sensitive K2P channels. For example, TRAAK channels may be usually co-expressed with TREK-1 and/or TREK-2 channels so that riluzole displayed a net effect of inhibition on temperature-sensitive leak K^+^ currents. Another possibility is that temperature-sensitive K2P channels in these neurons may be formed as heteromeric channels by TRAAK, TREK-1, and/or TREK-2 [3]. These heteromeric channels are shown to be functional in heterologous expression system but their pharmacological profiles have not been well characterized. The contribution of each subtype of these channels to the temperature sensitive leak K^+^ currents in DRG neurons will rely on subtype selective blockers in future studies.

Thermal K2P channels have been suggested to be involved in controlling nociceptive neuron excitability and may be therapeutic targets for treating pathological pain [2]. Thermal K2P channel expression can be downregulated following inflammation, which may be an underlying mechanism of enhanced neuronal excitability [17]. Functions of thermal K2P channels may be downregulated by G-protein coupled signaling molecules [8], and the inhibitory effects on temperature-sensitive leak K^+^ currents by PGF2α are consistent with this regulatory mechanism. Functional downregulation of leak K^+^ channels in nociceptive neurons may sensitize nociceptors to induce pathological pain. On the other hand, K2P channel activators may decrease nociceptors’ excitability and may be used to alleviate pathological pain. Efforts have been put recently to synthesize selective K2P channel activators for potential therapeutic uses for pain management [5, 19].

## Abbreviations

DRG: Dorsal root ganglion
K2P: Two-pore domain K^+^ channels
PBS: Phosphate Buffered Saline
PGF2α: Prostaglandin F2α
